# Isolation of Highly Pathogenic Avian Influenza H5N1 Virus from Saker Falcons (*Falco cherrug*) in the Middle East

**DOI:** 10.1155/2009/294520

**Published:** 2008-11-24

**Authors:** Henju Marjuki, Ulrich Wernery, Hui-Ling Yen, John Franks, Patrick Seiler, David Walker, Scott Krauss, Robert G. Webster

**Affiliations:** ^1^Division of Virology, Department of Infectious Diseases, St. Jude Children's Research Hospital, Memphis, TN 38105, USA; ^2^Central Veterinary Research Laboratory, P.O. Box 597, Dubai, United Arab Emirates; ^3^Department of Pathology, University of Tennessee, Memphis, TN 38105, USA

## Abstract

There is accumulating evidence that birds of prey are susceptible to fatal infection with highly pathogenic avian influenza (HPAI) virus. We studied the antigenic, molecular, phylogenetic, and pathogenic properties of 2 HPAI H5N1 viruses isolated from dead falcons in Saudi Arabia and Kuwait in 2005 and 2007, respectively. Phylogenetic and antigenic analyses grouped both isolates in clade 2.2 (Qinghai-like viruses). However, the viruses appeared to have spread westward via different flyways. It remains unknown how these viruses spread so rapidly from Qinghai after the 2005 outbreak and how they were introduced into falcons in these two countries. The H5N1 outbreaks in the Middle East are believed by some to be mediated by wild migratory birds. However, sporting falcons may be at additional risk from the illegal import of live quail to feed them.

## 1. Introduction

Infection of birds of prey with highly pathogenic avian influenza (HPAI) has been reported only in isolated cases. In 2000, Manvel et al. reported the discovery of HPAI H7N3 isolated from a peregrine falcon [1]. Another HPAI virus of the H7 subtype was found in a Saker falcon during an outbreak in poultry in Italy in 2000 [2]. Hong Kong has had a series of cases. In January 2004, Hong Kong authorities confirmed that a peregrine falcon had died of H5N1 infection [3, 4]. In March 2006, HPAI H5N1 infections were discovered in another peregrine falcon [5]. Most recently, in March 2008, a wild peregrine falcon found sick was confirmed to be infected with H5N1 virus [6]. In Belgium, 2 crested hawk eagles (*Spizaetus nipalensis*) smuggled from Thailand for a commercial raptor breeding farm were discovered to be infected with HPAI H5N1 virus and were killed by authorities [7]. Kuwait reported 39 confirmed cases of H5N1 infection in February 2007; 20 infected birds were falcons at a zoo and a farm in Southern Kuwait, and the remainder were domestic birds caged outdoors [8, 9]. The first confirmed case of lethal HPAI H5N1 infection in Saudi Arabia was reported in January 2006 in a Saker falcon [8]. In November 2007, an H5N1 outbreak killed 1500 chickens at a farm in the Al-Kharj region of Saudi Arabia, about 150 kilometers South of Riyadh [8]. No human cases have been reported to date in Saudi Arabia or Kuwait.

The increasing number of falcons infected with HPAI H5N1 viruses demonstrates their susceptibility to these pathogens. Because many falcon species are migratory or occupy extensive territories, infected falcons may contribute to the spread of avian influenza viruses within or between countries. The best-studied falcon species are the peregrine falcon (*Falco peregrinus*) and the Saker falcon (*Falco cherrug*), which feed exclusively on live, medium-sized birds, such as pigeons, shorebirds, waterfowl, and songbirds [10, 11]. The susceptibility of some of these prey species to HPAI H5N1 infection [12] increases the risk of virus transmission to falcons. Peregrine falcons, which can fly and dive at high speed, have long been used in the sport of falconry in the Middle East. Although there is still no direct evidence of virus transmission from falcons to humans, the practice of falconry brings birds of prey into close contact with humans, thereby increasing the risk of transmission to humans or to poultry.

It is not known how HPAI H5N1 virus spread so rapidly westward after the 2005 outbreak at Qinghai Lake, Western China, or how falcons in the Middle East contracted HPAI H5N1 virus. To acquire information that may answer these questions, we characterized two HPAI H5N1 viruses isolated from dead female Saker falcons in the region: A/Falcon/Saudi Arabia/D1795/2005 (Fa/SA/05) and A/Falcon/Kuwait/D286/2007 (Fa/KW/07). The falcon that died in Saudi Arabia was believed to have been in the country for the past 2 years, and it seems highly likely that the falcon had been kept in captivity during the entire period. The falcon had lost its appetite and was passing green feces during its last 2 days of life. It was confirmed to have died of H5N1 avian influenza virus [8]. Unfortunately, there is no information available about the falcon found dead in Kuwait. Organs removed from the two falcons were sent to the Central Veterinary Research Laboratory in Dubai, United Arab Emirates (UAE), in November 2005 and February 2007, respectively.

## 2. Materials and Methods

### 2.1. Viruses and Cells

The H5N1 isolates Fa/SA/05 and Fa/KW/07 were obtained from the Central Veterinary Research Laboratory, Dubai, UAE, and stored in the influenza virus repository of St. Jude Children's Research Hospital. All experiments were conducted in a USDA-approved biosafety level (BSL) 3+ containment facility. Stock viruses were prepared by inoculating field samples into the allantoic cavities of 10-day-old embryonated chicken eggs and incubating them at 35°C for 48 hours. Aliquots of stock viruses were stored at −80°C. Madin-Darby canine kidney (MDCK) cells were obtained from the American Type Culture Collection (Manassas, Va, USA) and maintained in Dulbecco's Modified Eagle's Medium (DMEM) supplemented with 10% fetal calf serum and 1% antibiotics (penicillin/streptomycin).

### 2.2. Infectivity

The 50% tissue culture infectious dose (TCID_50_) was determined in MDCK cells after incubation at 37°C for 3 days, and the 50% egg infectious dose (EID_50_) was determined in 10-day-old embryonated chicken eggs after 40 hours of incubation at 35°C. TCID_50_ and EID_50_ values were calculated by the method of Reed and Muench [13].

### 2.3. Sequencing and Phylogenetic Analysis of Influenza Virus Genes

Viral RNA was isolated from virus-containing allantoic fluid with the RNeasy Mini kit (Qiagen, Valencia, Calif, USA), and one-step RT-PCR (Qiagen) was done with a universal primer set [14]. PCR products were purified by using the QIAquick PCR purification and gel extraction kits (Qiagen). Sequencing was performed by the Hartwell Center for Bioinformatics and Biotechnology at St. Jude using BigDye Terminator (v.3; Perkin Elmer Applied Biosystems, Foster City, Calif, USA) chemistry and synthetic oligonucleotides. Phylogenetic analysis was based on the HA and NA genes. Selected HA and NA sequences from H5N1 viruses isolated after 1996/1997 were downloaded from the Influenza Sequence Database at Los Alamos National Laboratory (http://www.flu.lanl.gov/) [15]. Sequences were compared by using the ClustalW alignment algorithm with the BioEdit Sequence Alignment Editor. To estimate phylogenetic relationships, nucleotide sequences were analyzed by the neighbor-joining method with 100 bootstraps by using the phylogeny inference package (PHYLIP) version 3.65.

### 2.4. Pathogenicity in Chickens, Ducks, and Mice

All animal studies were conducted under applicable laws and guidelines and after approval from the St. Jude Animal Care and Use Committee. The pathogenicity of the two falcon H5N1 isolates in chickens was determined in groups of 3 (Fa/SA/05, stock virus concentration: 10^8.769^ EID_50_/mL) or 10 (Fa/KW/07, stock virus concentration: 10^9.231^ EID_50_/mL) 6-week-old, specific-pathogen-free white leghorn chickens (*Gallus gallus domesticus*) by intravenous injection with 0.1 mL of 10-fold dilutions of virus-containing allantoic fluid. The chickens were examined daily for 10 days for clinical signs of disease and for mortality. The experiment was stopped after the birds found dead within 24 hours postinoculation (p.i.).

Pathogenicity in mallards was determined as described previously [16]. Groups of five 5-week-old Mallard ducks (*Anas platyrhynchos*) were inoculated with 10^6^ EID_50_ of virus in a 1-mL volume (0.3 mL each was applied to the trachea and the throat, and 0.2 mL each to nares and eyes). All birds were observed daily for clinical signs and mortality over a 10-day period. Tracheal and cloacal swabs were collected every other day starting on day 3 p.i., and viral titers (TCID_50_) were determined.

The pathogenicity of the two falcon H5N1 isolates in mammals was determined in female 6-week-old BALB/c mice. Groups of mice (*n* = 3) were inoculated intranasally with 100 plaque-forming units (PFUs) of virus in 50 *μ*L of PBS. Brain, lungs, liver, spleen, and blood were collected from 3 mice in each group on days 3, 6, and 9 p.i. to evaluate organ tropism. Organs were homogenized in 1 mL of PBS, and virus was titrated (log_10_ TCID_50_/mL) in MDCK cells.

### 2.5. Hemagglutinin Inhibition (HI) Assay Using H5N1 Ferret Antisera

Ferrets obtained through the ferret breeding program at St. Jude or from Marshall Farms (North Rose, NY, USA) were intranasally inoculated with 10^6^ EID_50_ of virus in 1 mL of PBS under isoflurane anesthesia. Serum was collected on day 21 p.i. or 10 days after a boost dose of 1 mL of a 1 : 10 dilution of stock virus in PBS by footpad injection or intranasal inoculation. The antisera were treated with receptor-destroying enzyme and used for HI assay as previously described by Palmer et al. [17].

## 3. Results

### 3.1. Antigenic Characterization

We assessed the relation of the falcon H5N1 influenza viruses to other H5N1 viruses by hemagglutinin inhibition (HI) assay with a panel of reference sera collected from ferrets challenged with various H5N1 isolates representing clades 1, 2.2, and 2.3. The two falcon H5N1 isolates had slightly different reactivity patterns (see Table 1). Both viruses had relatively high HI titers (160 for Fa/SA/05 and 320 for Fa/KW/07) against A/chicken/Pakistan (Lahore)/NARC2411.A/06 (clade 2.2) antiserum, suggesting that they are closely related to the H5N1 virus from that region and to viruses of clade 2.2. The Fa/KW/07 virus was not highly reactive with antisera generated against Fa/SA/05, suggesting that the two isolates differ antigenically despite their common origin.

### 3.2. Molecular Characterization

There were 4 amino acid differences in their HA proteins at positions 12, 178, 512, and 553. The amino acid at position 178 is located close to the HA receptor binding sites (H5 HA numbering). The HA proteins of both viruses contained multiple basic amino acids at the connecting peptide between HA_1_ and HA_2_ (PQGERRRKKR at positions 321–330), which is a characteristic of influenza viruses that are highly pathogenic in chickens [18, 19]. Interestingly, the HA cleavage site of the two falcon H5N1 viruses differed in a single basic amino acid (R>G) from that of their ancestor viruses Gs/Gd/1/96 and Ck/HK/220/97 and from that of H5N1 viruses isolated in 2004 in Thailand, Indonesia, Vietnam, and Eastern China (PQ$\underline{\text{R}}$ERRRKKR).

We also analyzed the amino acids of the polymerase genes. A change at position 627 of the PB2 protein from glutamic acid (E) to lysine (K) was required for the high virulence of the 1997 H5N1 viruses in mice [20, 21] and for that of H7N7 viruses in humans [22]. Sequence analysis revealed a lysine at that position in both isolates, suggesting that both are lethal to mice. The PB1 and PA proteins of the two H5N1 isolates possessed tyrosine at position 436 and threonine at position 515. In contrast, the T515>A amino acid change in PA and the Y436>H change in PB1 were previously shown to abrogate the pathogenicity of H5N1 constructs in ducks, and the Y436>H mutation in PB1 reduced transmission efficiency in ducks. The T515>A mutation in PA eliminated virus lethality in ducks but not in mice and ferrets [16].

### 3.3. Potential Sensitivity to Antiviral Treatment

Amantadine-resistant influenza A variants carry amino acid substitutions at residues 26, 27, 30, 31, or 34 of the M2 protein [23, 24]. No substitutions at these residues were identified by sequence analysis in either H5N1 virus (see Table 2), suggesting that both are potentially sensitive to M2 inhibitors. Mutations in conserved NA framework or catalytic residues (amino acid residues 119, 274, 292, and 294, N2 numbering) may reduce virus sensitivity to NA inhibitors [25]. No substitutions were observed in the conserved NA residues of the studied H5N1 viruses (see Table 2), suggesting that they may be sensitive to neuraminidase inhibitors.

### 3.4. Phylogenetic Analysis

**Figure 1:**
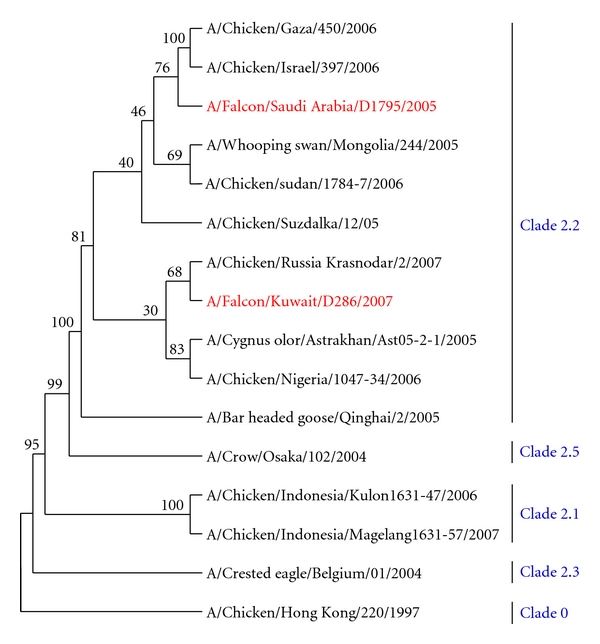
Phylogenetic relationships based on the HA (nucleotides 1–1684) genes of the Fa/SA/05 and Fa/KW/07 viruses. The nucleotide sequences were analyzed by PHYLIP 3.65 software using the neighbor-joining method with 100 bootstraps. The tree was rooted to the HA gene of A/Chicken/Hong Kong/220/97. Viruses in bold red type were characterized in this study. Clades are indicated.

The HA and NA phylogenetic trees were rooted to the HA and NA genes of A/Chicken/Hong Kong/220/97 virus. The HA gene of the Fa/SA/05 virus was phylogenetically closely related to that of viruses isolated in 2006 from chickens in Israel and Gaza (clade 2.2), whereas the HA gene of Fa/KW/07 was closely related to that of a virus isolated in 2007 from chickens in Russia (clade 2.2) (see Figure 1). On the basis of this analysis, both were assigned to subclade 2 of clade 2 of H5 HA. The viruses clustered phylogenetically with 2005–2007 isolates from Asia, Africa, and the Middle East (see Figure 1).

**Figure 2:**
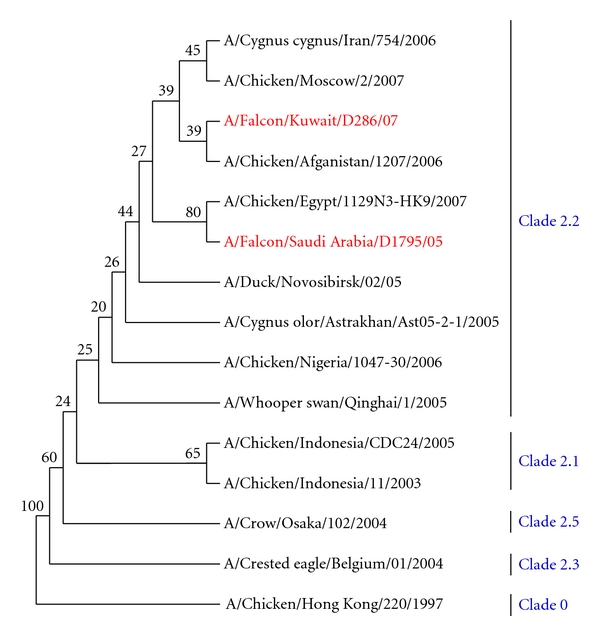
Phylogenetic relationships based on the NA (nucleotides–1388) genes of Fa/SA/05 and Fa/KW/07 viruses. The nucleotide sequences were analyzed by PHYLIP3.65 software using the neighbor-joining method with 100 bootstraps. The NA phylogenetic tree was rooted to the NA gene of A/Chicken/Hong Kong/220/97. Viruses in bold red type were characterized in this study. Clades are indicated.

The general topology of the N1 tree differs from that of the HA tree because of differences in stalk length. There is a 20-amino acid deletion in the stalk region (positions 49 to 68) in the current 2003–2007 and 2001-2002 China poultry isolates. A 19-amino acid deletion (positions 50 to 68) was observed in the H5N1/97 Hong Kong human and poultry isolates. Both of the falcon H5N1 viruses had a 20-amino acid deletion in the stalk region of NA. The NA gene of the Fa/SA/05 virus was phylogenetically closely related to that of A/chicken/Egypt/1129N3-HK9/2007 (clade 2.2), whereas the NA gene of Fa/KW/07 was phylogenetically closely related to that of A/chicken/Afghanistan/1207/2006 (clade 2.2) (see Figure 2).

### 3.5. Organ Tropism in a Mouse Model

**Figure 3:**
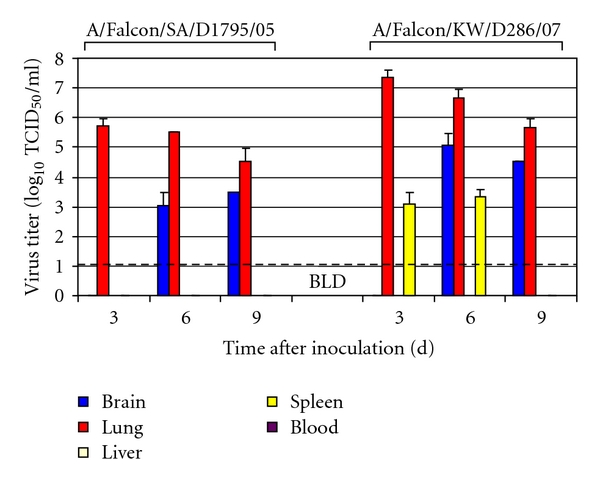
Mean titers of Fa/SA/05 and Fa/KW/07 viruses in mouse organ homogenates. Mice were inoculated with 100 PFU of each stock virus. Dashed line indicates detection limit (10 TCID_50_/mL). BLD: below level of detection. Error bars are SD obtained from mice (*n* = 3) killed at various times.

To determine the pathogenicity of the two falcon H5N1 isolates in a mammalian host, we inoculated BALB/c mice intranasally and measured virus replication in the brain, lungs, liver, spleen, and blood. Inoculation with 100 PFU of Fa/KW/07 virus resulted in high virus titers (mean 10^7.35^ TCID_50_/mL) in the lungs on day 3 p.i.; titers had declined slightly on days 6 and 9 p.i. (see Figure 3). Virus titers of Fa/SA/05 virus were approximately 1 log lower than those of Fa/KW/07 on all days studied. On day 6 p.i., the mean Fa/KW/07 virus titer in the brain was 10^5.07^ TCID_50_/mL and that of Fa/SA/05 was 10^3.02^ TCID_50_/mL (see Figure 3). Virus was detected in the spleens of mice inoculated with Fa/KW/07 virus only 3 and 6 days p.i. All mice inoculated with either falcon H5N1 virus died by day 9 p.i.

### 3.6. Virulence and Pathogenicity in Avian Species

To determine whether the two falcon H5N1 isolates retained pathogenicity for avian species, we inoculated 6-week-old, specific-pathogen-free white leghorn chickens by intravenous injection. Both viruses caused 100% mortality within 24 hours.

**Figure 4:**
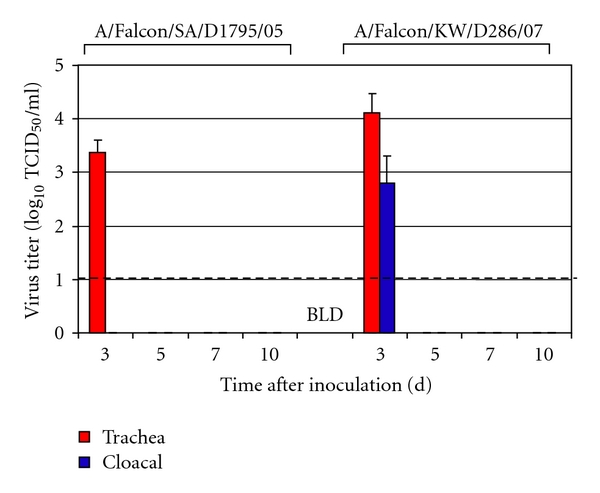
Mean (±SE) tracheal and cloacal virus titers in mallard ducks inoculated with Fa/SA/05 and Fa/KW/07 viruses. Ducks (*n* = 5) were inoculated with 10^6^ EID_50_ of each stock virus. Virus was tittered from tracheal and cloacal swabs collected 3 days after inoculation to determine the TCID_50_. Dashed line indicates detection limit (10 TCID_50_/mL). BLD: below level of detection.

We then inoculated 2 groups of 5 mallard ducks via natural routes (trachea, throat, nares, and eyes) with 10^6^ EID_50_ of Fa/SA/05 and Fa/KW/07 stock virus. All ducks survived during the 10-day observation period. Clinical signs (cloudy eyes and ataxia) were observed in 2 of 5 ducks on day 5 after inoculation with Fa/KW/07. Fa/SA/05 caused only cloudy eyes in 1 of 5 ducks during days 5 to 7 p.i. Tracheal and cloacal swabs were collected every other day starting on day 3 p.i. Both viruses were shed from the trachea on day 3 p.i. (see Figure 4), and Fa/KW/07 virus was detected in cloacal samples on day 3 p.i. No virus was detected in specimens obtained on days 5, 7, and 10 p.i.

## 4. Discussion

Accumulating data demonstrate that falcons are extremely susceptible to HPAI H5N1 infection. We investigated the molecular, phylogenetic, antigenic, and pathogenic properties of 2 H5N1 viruses isolated from dead falcons in the Middle East. Phylogenetic and antigenic analyses indicated that both viruses originated from Qinghai-like H5N1 viruses that caused a massive die-off among wild birds in China in 2005. However, it is still not clear how these viruses were introduced into the region, and how the falcons in the affected countries contracted the HPAI H5N1 virus.

There are several possibilities that may explain the introduction of these viruses. Since no cases of H5N1 infection were reported in the Middle East before 2005, the first possibility is that the virus was introduced by wild birds migrating westward after the 2005 outbreak in China. The virus rapidly spread westward through various regions, including the Middle East [26], and subsequently spread to domestic poultry and probably to other avian species, including falcons.

A second possibility is that the virus was introduced by infected falcons illegally imported into the Middle East from China, Mongolia, or Russia. Thousands of wild-caught falcons are traded on the black market withoutveterinary inspection, licensing from the Convention on International Trade in Endangered Species of Wild Fauna and Flora, or border controls [27]. Illegal trading of falcons can contribute to the global spread and transmission of HPAI viruses, and therefore export/import activity should be strictly regulated. As an example, falcons brought into the UAE must be quarantined for 21 days; blood and nasal mucus are tested during the first and second weeks for H5N1 virus [28]. These extraordinary security measures have so far prevented any cases of HPAI H5N1 infection in falcons in the UAE.

A third possibility is that the falcons were infected via diseased pigeons and/or quail, which are their normal food source. To feed hunting falcons, tens of thousands of live quail areflown from the Arabian Gulf into royal falconry camps across Central Asia without veterinary inspection [27]. Many falcons in North America, Germany, and the Persian Gulf area have died after feeding on birds (especially pigeons and quail) and other prey infected with avian influenza viruses [29]. Therefore, falcons in captivity should be fed only birds that have been inspected by a veterinarian.

The phylogenetic tree of H5 HA revealed that although the two falcon H5N1 viruses originated from the same ancestor viruses (clade 2.2, Qinghai-like), they are likely to have spread westward via different flyways. Because the HA of Fa/SA/05 shares strong sequence similarity with the HAs of 2006 chicken isolates from Gaza and Israel, this virus appears to have spread from Qinghai Lake toward the African continent via a “southern” flyway. In contrast, the HA of Fa/KW/07 is closely related to that of a 2007 chicken isolate from Russia, suggesting that the Kuwaiti isolate was carried toward Europe via a “northern” flyway. The two viruses showed slightly different reactivity patterns with antisera from ferrets inoculated with various H5N1 viruses. The fact that the Fa/KW/07 virus was poorly reactive with antisera is generated against the Fa/SA/05 virus (see Table 1), suggesting that it may have undergone minor antigenic drift in its HA gene. In addition, the fourfold difference between the homologous and heterologous titer also suggests only minor antigenic differences. Therefore, based on this observation, an indication of different origin of isolates should be predicated on the HA phylogenetic analysis rather than on the slight antigenic differences of the two falcon isolates. Furthermore, sequence analysis revealed a 20-amino acid deletion in the NA stalks of both viruses; this feature is a characteristic of chicken-adapted influenza viruses.

Interestingly, although both falcon H5N1 isolates were uniformly lethal to chickens, their replication was limited in mallard ducks. Only about one third of inoculated ducks showed clinical signs of illness, and virus was shed only on day 3 p.i. (see Figure 4). This low pathogenicity may allow ducks to passively carry and shed these and other HPAI H5N1 viruses.

The Saudi Arabian and Kuwaiti falcon H5N1 viruses were replicated in multiple mouse organs, particularly brain and lungs, without prior adaptation (see Figure 3). H5N1 influenza virus lethality and spread to the brain in mice usually require multiple passages for adaptation [30]. The high lethality of these isolates in mice is probably related to the K627 residue of the PB2 protein [20, 21]. Interestingly, the PB2s of all H5N1 viruses isolated from humans and those of all avian H5N1 isolates from the recent outbreaks in Qinghai Lake (Western China), Russia, and Nigeria, possess lysine (K) at position 627 [21, 31], whereas the PB2s of their progenitor avian viruses contain glutamic acid (E) at this position.

All subtypes of influenza A virus have the potential to become pandemic strains, but the currently circulating HPAI H5N1 viruses are of greatest concern. Our study provides the first molecular and biological insights into the HPAI H5N1 viruses isolated from dead falcons in the Middle East. This information will be useful in determining which measures should be taken to provide effective surveillance and protection of these birds in the affected countries and around the globe. During the preparation of this manuscript, Lierz et al. [31] reported that falcons survived HPAI H5N1 infection after vaccination with inactivated H5N2 virus. The vaccinated birds did shed virus but at reduced titers. This approach may provide a partial solution to the protection of falcons in captivity.

## Figures and Tables

**Table 1:** Antigenic analysis of Fa/SA/05 and Fa/KW/07 hemagglutinin using ferret polyclonal antisera. <: HI antibody titer less than 40. The HI titer for the homologous virus and antiserum is indicated by bold/underline. Viruses: HK213 (A/Hong Kong/213/03), VN1203 (A/Vietnam/1203/04), CkPak (A/Chicken/Pakistan(Lahore)/NARC/3320/4/06), FaSA (A/Falcon/Saudi Arabia/D1795/05), FaKW (A/Falcon/Kuwait/D286/06), JweHK (A/Japanese White Eye/Hong Kong/1038/06), and dkLao25 (A/Duck/Laos/25/06).

		HI titer against ferret antiserum to						
Clade	Virus	rgHK213	rgVN1203	CkPak	FaSA	FaKW	JweHK	dkLao25
1	rgHK213	$\underset{¯}{320}$	320	1280	640	160	640	80
1	rgVN1203	<	$\underline{160}$	40	80	<	<	<
2.2	CkPak	<	<	$\underset{¯}{320}$	160	640	<	<
2.2	FaSA	<	<	160	$\underline{160}$	160	<	<
2.2	FaKW	<	<	320	40	$\underset{¯}{320}$	40	<
2.3	JweHK	160	80	80	40	40	$\underset{¯}{320}$	640
2.3	dkLao25	<	<	<	40	<	<	$\underline{160}$

**Table 2:** Amino acid (aa) residues associated with virus sensitivity and resistance to antiviral drugs.

Protein	M2					NA			
aa position	**26**	**27**	**30**	**31**	**34**	**119**	**274**	**292**	**294**
Sensitive	L	V	A	S	G	E	H	R	N
Resistant	F	A/T/S/G	V/T/S	N/G	E	V/G/A/D	Y	K	S
Fa/SA/05	L	V	A	S	G	E	H	R	N
Fa/KW/07	L	V	A	S	G	E	H	R	N
